# YDJC Induces Epithelial-Mesenchymal Transition via Escaping from Interaction with CDC16 through Ubiquitination of PP2A

**DOI:** 10.1155/2019/3542537

**Published:** 2019-08-07

**Authors:** Eun Ji Kim, Mi Kyung Park, Gyeoung-Jin Kang, Hyun Jung Byun, Hyun Ji Kim, Lu Yu, Boram Kim, Hee-Sung Chae, Young-Won Chin, Jae Gal Shim, Ho Lee, Chang Hoon Lee

**Affiliations:** ^1^Pharmaceutical Biochemistry, College of Pharmacy, Dongguk University, Seoul, Goyang, 04620, Republic of Korea; ^2^Graduate School of Cancer Science and Policy, National Cancer Center, Goyang, 10408, Republic of Korea

## Abstract

Lung cancer is the number 1 cause of cancer-related casualties in the world. Appropriate diagnostic markers and novel targets for lung cancer are needed. Chitooligosaccharide deacetylase homolog (YDJC) catalyzes the deacetylation of acetylated carbohydrates; however, the role of YDJC in lung cancer progression has yet to be studied. A549 lung cancer orthotopic mouse model was used for mice experiments. We found that YDJC overexpression contributes to lung cancer progression in an orthotopic mouse model. Long-term treatment (48 h) induces YDJC expression in sphingosylphosphorylcholine (SPC)-induced epithelial-mesenchymal transition (EMT). Gene silencing of YDJC (siYDJC) reduced N-cadherin expression and increased E-cadherin expression in SPC-induced EMT. Overexpression of YDJC reverses them but overexpression of the deacetylase deficient mutant YDJC_D13A_ could not. Interestingly, overexpression of CDC16, a YDJC binding partner, suppressed EMT. ERK2 is activated in siCDC16-induced EMT. YDJC overexpression reduces expression of protein phosphatase 2A (PP2A), whereas CDC16 overexpression induces PP2A expression. YDJC overexpression induced ubiquitination of PP2A but YDJC_D13A_ could not. CDC16 overexpression increased the ubiquitination of YDJC. These results suggest that YDJC contributes to the progression of lung cancer via enhancing EMT by inducing the ubiquitination of PP2A. Therefore, YDJC might be a new target for antitumor therapy against lung cancer.

## 1. Introduction

Lung cancer represents the highest mortality rate among all cancers worldwide [1]. Unmet needs for lung cancer are very high, but the lack of diagnostic markers and druggable targets for controlling the progression of lung cancer has become a bottleneck in the development of anticancer agents.

Chitooligosaccharide deacetylase homolog (YDJC) is a member of the YDJC family. YDJC carries out the deacetylation of the acetylated moiety in carbohydrates, an essential step in the decomposition of oligosaccharides although the exact substrate of YDJC in mammals has been identified [2]. Genetic approaches informed that the YDJC gene is related to several diseases such as ulcerative colitis, psoriasis, and Crohn's disease [3, 4]. It was reported as one of the target genes induced by mTOR activation [5]. However, the function of YDJC in lung cancer progression is unknown although we published the first report about YDJC's role in keratin reorganization which is related to the changes of viscoelasticity in metastatic cancer cells [6].

Epithelial-mesenchymal transition (EMT) is considered the first step in lung cancer progression [7]. In the course of EMT, epithelial cancer cells in original tumor sites lose cell-cell contact and apicobasal polarity but gain the power for migration and invasion to adjacent tissues [8]. Although EMT facilitates cancer cell motility, EMT impacts the overall hallmarks of cancer, including proliferation, resistance to therapeutics, cancer initiation, survival, cancer metabolism, and immune evasion [8, 9]. Recently, EMT is attracting the spotlight as a target to suppress immune evasion of cancer [10, 11].

The aim of this study is to clarify YDJC's role and molecular mechanism in the EMT process.

## 2. Materials and Methods

### 2.1. Materials

Welgene Inc. (Seoul, Korea) supplied the Roswell Park Memorial Institute (RPMI)-1640 medium, fetal bovine serum (FBS), phosphate-buffered saline (PBS), and penicillin and streptomycin (PS). Lipofectamine™ 2000 or JetPEI was supplied by Invitrogen (Carlsbad, CA, USA) or Polyplus-transfection (Illkirch-Graffenstaden, France), respectively. Sphingosylphosphorylcholine (SPC) was procured from Matreya (Pleasant Gap, PA, USA). Calbiochem (La Jolla, CA, USA) supplied the MAP kinase inhibitors such as PD98059, SP600125, and SB203580. Mouse monoclonal antibodies specific for E-cadherin (610181, 1:1000), N-cadherin (610920, 1:1000), and PP2A (610555, 1:2000) were from BD Biosciences (San Jose, CA, USA). The anti-YDJC (ab107873, 1:500) antibody was supplied by Abcam (Cambridge, MA, USA). Cell Signalling Technology (Beverly, MA, USA) provided the phosphospecific antibody to detect ERK1/2 (#9102, 1:1000) and anti-ERK (#9106, 1:1000) antibodies. Anti-ubiquitin, anti-CDC16 (sc-365636, 1:1000), and anti-*β*-actin (sc-47778, 1:5000) antibodies were provided by Santa Cruz Biotechnology, Inc. (Santa Cruz, CA, USA). Molecular Probes, Inc. (Eugene, OR, USA) supplied Alexa Fluor 594 goat anti-mouse and Alexa Fluor 488 goat anti-rabbit antibodies. All of these reagents were freshly made at the moment of each experiment.

### 2.2. Cell Culture

The American Type Culture Collection (Manassa, VA, USA) supplied the A549, H838, and H1299 lung cancer cell lines. These cells were cultured at 37°C in 95% air and 5% CO_2_ in an RPMI-1640 media added to a 10% heat-inactivated FBS and PS solution (100 U/mL P, and 100 mg/mL S).

### 2.3. Western Blot and Quantitation of Band Density

Western blot was done, as mentioned before [6, 12]. After termination of the cell culture, the lung cancer cells were rinsed 2 times with ice-chilled PBS and broken in RIPA buffer containing Xpert phosphatase and protease inhibitor cocktails from GenDEPOT Inc. (Austin, TX, USA) on ice for 30 min. Lung cancer cell lysates were separated by centrifugation at 15,000 rpm for 15 min at 4°C. The protein amounts were measured using the Bradford method. Proteins (20–30 *μ*g) in the lung cancer cell lysate were resolved by 8–12% gradient sodium dodecyl sulfate-polyacrylamide gel electrophoresis (SDS-PAGE) and transferred to a polyvinylidene fluoride membrane in the transfer buffer consisting of 192 mM glycine, 25 mM Tris-HCl (pH 8.8), and 10% v/v MeOH [6]. After blocking nonspecific sites with 5% nonfat dry milk, the membrane was treated with the primary antibody in 3% bovine serum albumin (BSA) PBS solution at 4°C overnight and then incubated for 1 h in a peroxidase-conjugated secondary antibody (1:5,000, Santa Cruz Biotechnology Inc.) PBS solution at room temperature (RT). Immunoreactive proteins were found by PowerOpti-ECL detection reagent from Animal Genetics Inc. (Gyeonggi, Korea) [6].

Determination of the Western band's density was achieved by ImageJ 1.52a (National Institutes of Health) and normalized to loading control. The results are representative data from 3 independent experiments.

### 2.4. Reverse Transcription-PCR (RT-PCR) and qRT-PCR

Total RNA was extracted using TRIzol reagent (Invitrogen) as described by the manufacturer, and RT-PCR was performed according to the instructions provided by Access RT-PCR Systems (Promega, Madison, WI, USA). To quantify the RNA expression levels, qRT-PCR was performed in a 384-well plate on a LightCycler® 480 II real-time PCR system (Roche Diagnostics, Mannheim, Germany) using SYBR Premix and Ex Taq from Takara Bio (Shiga, Japan) as described by the manufacturer and the data were normalized to expression of a control gene* GAPDH*. The cycle number at which the product level exceeded an arbitrarily selected threshold (CT) was decided for each target sequence, and the amount of RNA relative to control RNA was depicted using the formula 2^−ΔCT^, where ΔCT is the difference between the crossing point thresholds of a target gene versus* GAPDH*. The following primers were applied for qRT-PCR:* YDJC*, 5-GGAGACGTGGATTTGCCTCA -3′ and 5′-TAAAGCGACCCCATAGGC-3′;* CDC16*, 5′-ATGACCTCGACCAGCAACAG-3′ and 5′-GCTGCAAGGTAACGACATGC-3′;* PP2A-C*, 5′- ACGGAATGGCTAATCTGT-3′ and 5′- GATTGCTCTCCTCCACTAA-3′;* GAPDH*, 5′-GAAGGT GAAGGTCGGAGTC-3′ and 5′-GAAGATGGTGATGGGATTTC-3′.

### 2.5. Knockdown of a Gene by Small-Interfering RNA or Plasmid DNA Transfection

YDJC small-interfering RNA (siRNA) (sequence (I): 5′- GCUUCUUCCUUGGCAAG- AU-3′, (II): 5′- AUCUUGC- CAAGGAAGAAGC -3′), CDC16-1 siRNA (sequence (I): 5′-GGACGCUUGUAGAGCCU- GATT-3′, (II): 5′- UUCAGCUCUACAAGCGUCCTT-3′), CDC16-2 siRNA (sequence (I): CCGUGGGAUUUCAGGGAAUTT-3′, (II): 5′-AUUCCCUGAAAUCCCAC-GGTT-3′), ERK1 siRNA (sequence (I): 5′-CGGCCUAUGACCACGUGCGCATT-3′, (II) 5′-UGCGCACGUGGUCAUAGGCCGTT-3′), and ERK2 siRNA (sequence (I): 5′-GAGGAUUGAAGUAGAACAGtt-3′, (II): 5′-CUGUUCUACUUCAAUCCUCtt-3′) were supplied by ST Pharm (Seoul, Korea) [6]. Genes were knockdown in the lung cancer cells with each siRNA using Lipofectamine™ 2000 (Invitrogen) according to the manufacturer's protocol. The ratio of siRNA to Lipofectamine reagent was 1:1.5. For transient overexpression study, cDNA of YDJC was cloned into the pcDNA3.1 vector. 70~80% confluent cells were transfected with JetPEI reagent (Polyplus Transfection, San Marcos, CA ) according to the recommendations by manufacturer (DNA:JetPEI=1:2). 24~48 h after transfection, breast cells were used in further experiments.

### 2.6. Assay of Lung Cancer Cell Invasion

The lung cancer cell invasion experiment was done with Matrigel (0.5 *μ*g/mL) coated-Transwell inserts (Neuro Probe Inc., Gaithersburg, MD, USA), as mentioned before [6, 13]. Lung cancer cells (1×10^6^ cells/mL) resuspended in serum-free medium were put to the upper chamber of the insert. Each lower chamber was filled with a medium containing 10% FBS [6]. After 16 h incubation, cells that failed to invade were removed off the upper surface of the membrane. Lung cancer cells that had invaded to the lower surface were stained by Hema 3 from Fisher Scientific Inc. (Houston, TX, USA) and photographed, and the cell numbers in four randomly chosen fields were computed at 20× magnification [6]. All the experiments were repeated at least 3 times with 2 replicates each [6].

### 2.7. Assay of Lung Cancer Cell Migration

The lung cancer cell migration experiment was carried out in 10 *μ*g/mL fibronectin-coated Transwell inserts [6]. Lung cancer cells (1×10^6^ cells/mL) resuspended in serum-free medium were loaded to the upper chamber of each insert as mentioned before [14]. Each lower chamber was filled with a medium containing 3% FBS. After 6 h incubation, nonmigrating lung cancer cells were removed from the upper surface of the membrane. Lung cancer cells that migrated to the lower surface were detected with Diff-quick reagent. Migrated lung cancer cells in four randomly selected fields were counted at 20× magnification [6]. All the experiments were done at least 3 times with 2 replicates each [6].

### 2.8. Immunofluorescence Staining

Immunofluorescence staining was done, as described previously [6, 15]. A549 cells were cultured on coverslips and fixed in cold MeOH for 10 min at RT. Then the A549 cells were permeabilized with a 10 min rinse in 0.1% Triton X-100 solution, also at RT, followed by rinsing several times in PBS containing 0.1% Tween-20. Next, the A549 cells were blocked with 3% BSA in PBS at RT for 1 h [6]. E-cadherin and N-cadherin primary antibodies were incubated with cells grown on coverslips overnight at 4°C, after which the antibody was rinsed out 4 times in PBS containing 0.1% Tween-20. Species-specific secondary antibodies conjugated in goat anti-mouse IgG antibody (Alexa Fluor 488, 1:500; Molecular Probes) and chicken anti-rabbit IgG antibody (Alexa Fluor 594, 1:500; Molecular probes) were incubated with the cells on the coverslips for 1 h at RT followed by 4 rinses in PBS with 0.1% Tween-20. The final samples were analyzed on slides using confocal microscopy (Nanoscope, Daejeon, Korea).

### 2.9. Immunoprecipitation

Immunoprecipitation (IP) was done, as mentioned before [6, 16]. Lysates of A549 cells were treated with mouse monoclonal anti-PP2A, rabbit polyclonal anti-YDJC, or mouse polyclonal anti-CDC16 antibodies overnight at 4°C, respectively [6]. Protein A/G magnetic beads from Pierce (Rockville, IL, USA) were treated to each sample tube and reacted for 1 h at RT according to protocol. 50 *μ*L of each eluted sample was separated by SDS-PAGE and detected with Western blotting.

### 2.10. Construction of A549_YDJC_, A549_YDJC-D13A_, and A549_shYDJC_ Cell Lines

A549_YDJC_ and A549_YDJC-D13A_ cell lines were established using plasmid DNA containing YDJC or YDJC_D13A_, respectively. pcDNA3.1YDJC and pcDNA3.1YDJC_D13A_ were transfected into the A549 cells, and stable cell lines were chosen in 1 mg/mL G418 disulphate (Duchefa, Haarlem, Netherlands). A549_Con_ cells were established with the empty vector containing only the Neomycin resistance gene. A549_shYDJC_ was obtained using lentiviral shRNA for YDJC transfected into the A549 lung cancer cells using Puromycin as a selection marker.

### 2.11. Histology and Immunohistochemistry (IHC)

At the time of sacrificing, lung tissues were fixed with 10 % formalin overnight and dehydrated in ethanol, embedded in paraffin, and sectioned at 4 *μ*m using Thermo Scientific Rotary Microtome Microm HM355S (Thermo Fischer Scientific, Germany) followed by staining with hematoxylin and eosin. Each slide was treated with 0.3% H_2_O_2_ solution to inactivate endogenous peroxidase for 30 min at RT. Tissues were blocked in 5 % Goat Serum in PBS; after blocking, the mounted sections were immunostained with primary antibodies N-cadherin (1:100; ab18203; abcam), E-cadherin (1:100; 610181; BD Biosciences), PP2A (1:100; 610555; BD Biosciences), phospho-ERK1/2 (1:100; 9106; Cell Signalling Technology), and ERK1/2 (1:100; 9102; Cell Signalling Technology) in PBS containing 5% Goat Serum. The cells were incubated with a biotinylated secondary antibody solution for 1 h at RT. Detection of antibodies was performed by the avidin-biotin complex (ABC) method using the Vectastain Elite ABC kit (Vector Laboratories, Burlingame, CA). Serial sections were stained with the DAB peroxidase substrate kit from Vector Laboratories (Burlingame, CA) and hematoxylin. Sections were dehydrated and coverslipped with mounting solution from Vector Laboratories. Images were obtained with a Zeiss Axiophot microscope (Carl Zeiss, Jena, Germany). For quantification analysis, three fields were randomly chosen on each slide. Each tissue section was semiquantitatively scored according to the percentage of positive cells. Positive cells were quantified using NIH ImageJ software (http://rsb.info.nih.gov/ij/)

### 2.12. Orthotopic Mouse Model

A549_YDJC_ and A549_Con_ cells were directly introduced into the left lung of NOD/SCID mice (male; n=7/group) as depicted elsewhere [16]. A549 cells (10^6^) were mixed with 50 *μ*L PBS containing Matrigel (1:1) and put into the left lateral thorax, approximately 1.5 cm above the lower rib line just below the inferior border of the scapula. The mice were euthanized 8 weeks later; the lungs were isolated and fixed in 10% neutrally buffered formalin, followed by histological examination. The Institutional Animal Care and Use Committee of the Korean National Cancer Center inspected all the experimental animal procedures.

### 2.13. Statistical Analysis

Data are represented as the mean ± standard deviation (SD) of at least 3 independent experiments done in triplicate. Analysis of the data was done using Student's t-test. Significance levels were *∗*p<0.05, *∗∗*p<0.01, and *∗∗∗*p<0.001.

## 3. Results

### 3.1. Overexpression of YDJC Promotes Lung Cancer Progression in an Orthotopic Mouse Model

**Figure 1:**
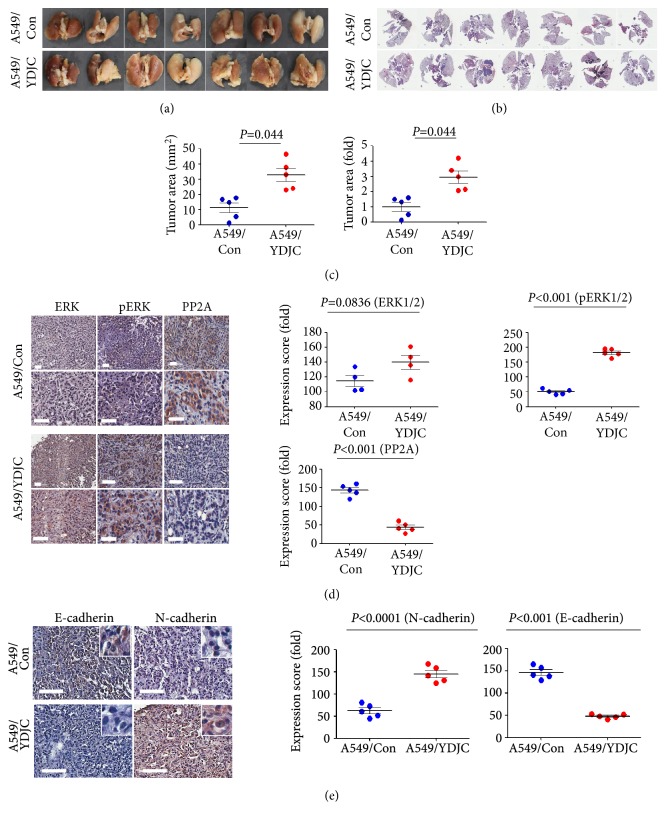
Overexpression of YDJC induces lung cancer progression in a lung cancer orthotopic mouse model. A549_YDJC_ and A549_Con_ cells were directly introduced via injection into the left lung parenchyma of NOD/SCID mice as described in the methods [16]. (a) Gross images of the lungs in NOD/SCID mice injected with A549_YDJC_ and A549_Con_ cells. (b) Hematoxylin and eosin staining of the lungs in NOD/SCID mice injected with A549_YDJC_ and A549_Con_ cells. Lungs were analyzed by hematoxylin and eosin staining. (c) Comparison of tumor areas: mice injected with A549_YDJC_ versus mice injected with A549_Con_ cells. Tumor areas were measured by intravital microscopic analysis (Axiotech Vario microscope, Zeiss, Germany). *∗*p<0.05 compared with the control group. (d) IHC staining for ERK, pERK, and PP2A. Scale bars, 20 *μ*m. (e) IHC staining for E-cadherin and N-cadherin. Scale bars, 20 *μ*m. In D and E, representative results are shown. Immunohistochemistry positive scores of ERK1/2, pERK1/2, and PP2A. Mean± SD; n=3. All* P*-values were based on two-tailed Student's t-test.

From our previous study, YDJC plays a significant role in SPC-induced keratin phosphorylation and reorganization through ERK activation [6]. However, in the prior report, we could not prove the role of YDJC in the* in vivo* mice models. Therefore, we examined the involvement of YDJC in lung cancer progression in an orthotopic mouse model. A549_YDJC_ and A549_Con_ cells were introduced into the lungs of NOD/SCID mice by injection. We found that YDJC overexpression increased the tumor area by 2.9 folds (*P*=0.044) in mice injected with A549_YDJC_ cells (Figures 1(a), 1(b), and 1(c)). Based on the previous report about the role of YDJC, we examined pERK and PP2A expression by IHC. pERK expression significantly increased, and PP2A expression reduced in the A549_YDJC_ cell-injected mice group compared with A549_Con_ cell-injected mice (Figure 1(d)).

Continuous phosphorylation and reorganization of keratin results in loss of keratin [17]. Interestingly, a recent study reported that loss of keratin 8 (K8), one of the epithelial cell-specific intermediate filament proteins, is one of the hallmarks of EMT [18]. So, we examined the expression of EMT markers, including E-cadherin (an epithelial marker) and N-cadherin (mesenchymal marker) in lung cancer tissues obtained from the orthotopic mouse model. E-cadherin expression was decreased more in tumor tissues from A549_YDJC_ than A549_Con_ injected groups (Figure 1(e)). By contrast, N-cadherin expression was induced higher in tumor tissues from A549_YDJC_ than A549_WT_ injected groups (Figure 1(e)). These results imply that YDJC expression might be related to EMT, which contributes to tumor progression [7].

### 3.2. YDJC Is Induced in SPC-Induced EMT of Lung Cancer Cells

**Figure 2:**
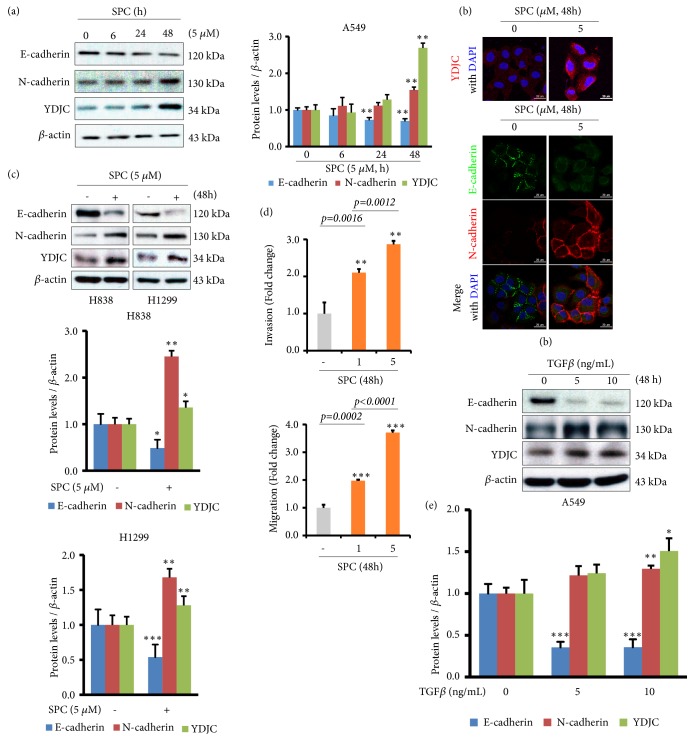
SPC induces EMT and YDJC expression is increased during SPC-induced EMT of lung cancer cells. (a) SPC time dependently induced the expression of the mesenchymal marker, N-cadherin, and YDJC and reduced the expression of E-cadherin in A549 lung cancer cells. (b) Confocal microscopic analysis of EMT markers and YDJC expression in SPC-induced EMT of A549 lung cancer cells. (c) SPC (5 *μ*M) induced the expression of the mesenchymal marker, N-cadherin, and YDJC and reduces the expression of E-cadherin in H838 and H1299 lung cancer cells. (d) SPC induced invasion and migration of lung cancer cells. After SPC treatment, lung cancer cells were put in the upper chamber of the Transwell insert. Then, the number of cells was calculated under four randomly selected high-power fields (20×). The results are a typical example of 3 independent experiments with similar results. *∗∗*p<0.01 and *∗∗∗*p<0.001 compared with the control group or the SPC-treated group. (e) TGF-*β*1 induced YDJC expression and EMT in A549 cells. In, (a), (c), and (e), the relative expression level of each set of protein was quantified and adjusted to that of *β*-actin with NIH Image software.

Loss of E-cadherin expression and gain of N-cadherin expression were observed in tumor tissues from the A549_YDJC_ injected groups (Figure 1(e)). Therefore, we were interested in the role of YDJC expression in EMT and investigated the link between YDJC and EMT via long-term SPC treatment in lung cancer cells. First, we determined whether SPC induces EMT. SPC (5 *μ*M) induced downregulation of E-cadherin and upregulated expression of N-cadherin and YDJC in a time-dependent manner (Figure 2(a)). This was also confirmed by confocal microscopy (Figure 2(b)). SPC-induced changes in EMT markers were also observed in H838 and H1299 lung cancer cells (Figure 2(c)). From the previous report, we know that 1 h SPC pretreatment can induce migration and invasion of lung cancer cells. We wonder whether 48 h SPC treatment also induces migration and invasion of lung cancer cells. 48 h SPC treatment also increased migration and invasion of A549 cells (Figure 2(d)). YDJC expression was also induced in TGF-*β*1-induced EMT (Figure 2(e)).

### 3.3. YDJC Is Involved in SPC-Induced EMT and the Deacetylase Activity of YDJC Is Essential in YDJC-Involved EMT

**Figure 3:**
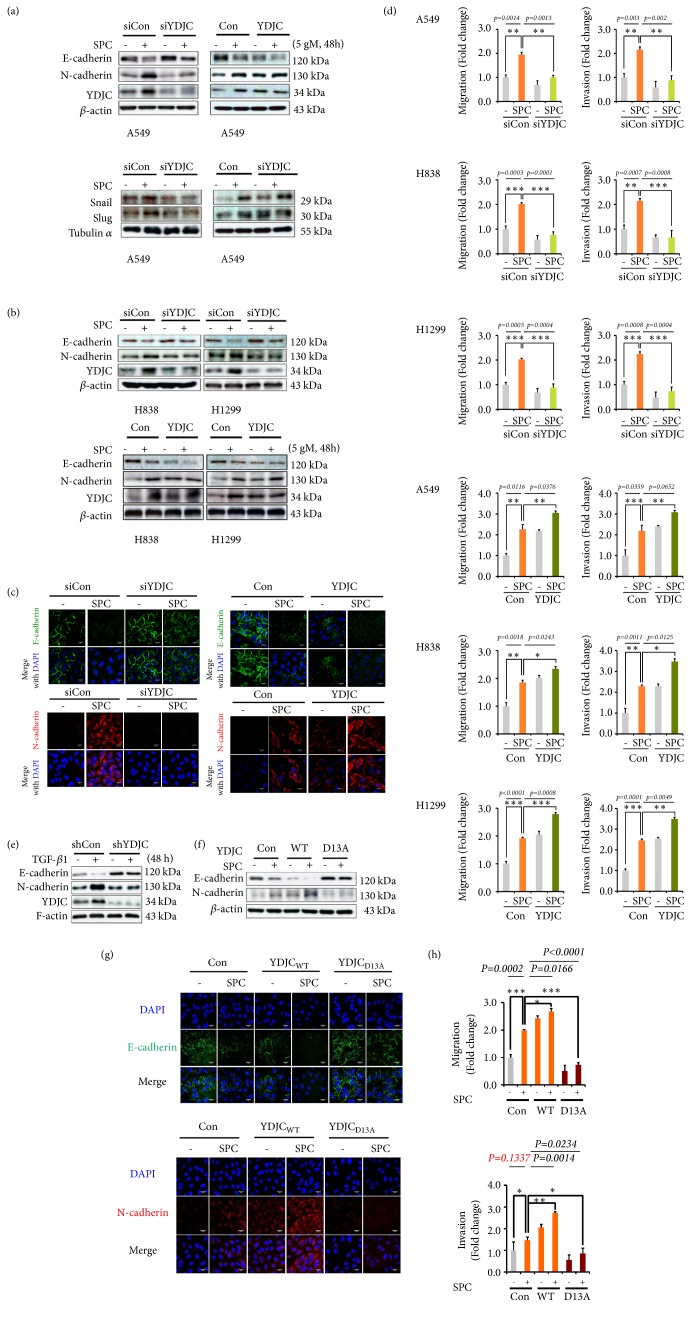
The deacetylase activity of YDJC promotes EMT of lung cancer cells. (a) YDJC siRNA suppresses and YDJC overexpression promotes SPC-induced EMT in A549 lung cancer cells. (b) YDJC siRNA suppressed SPC-induced EMT, and YDJC overexpression promoted EMT in H838 and H1299 lung cancer cells. (c) Confocal microscopic analysis of EMT markers and YDJC in SPC-induced EMT of A549 lung cancer cells. (d) YDJC siRNA suppressed and YDJC overexpression promoted SPC-evoked migration and invasion in A549, H838, and H1299 lung cancer cells. (e) TGF-*β*1 was unable to induce changes of EMT markers such as E-cadherin and N-cadherin in YDJC stably knockdown A549 lung cancer cells (A549_shYDJC_). In (a)-(d), the YDJC gene was silenced by transfecting the A549, H838, and H23 cells with the YDJC siRNA or control siRNA followed by stimulation with or without 5 *μ*M SPC for 48 h. (f) Effects of YDJC deacetylase activity on SPC-induced E-cadherin and N-cadherin expression. The overexpression of plasmids containing YDJC and YDJC_D13A_ (a dominant negative form of deacetylase) cDNA were analyzed by transfecting A549 cells with the plasmid containing YDJC or YDJC_D13A_ and a control empty vector (4 *μ*g) followed by treatment with SPC (5 *μ*M) for 48 h. (g) Confocal microscopic analysis of the effects of the deacetylase activity of YDJC on SPC-induced E-cadherin and N-cadherin expression. Effects of the deacetylase activity of YDJC on SPC-promoted migration and invasion. In (e) and (f), cell lysates were analyzed by Western blotting. YDJC gene overexpression was analyzed by transfecting A549, H838, and H1299 cells with a plasmid having YDJC or an empty vector (4 *μ*g) and subsequent treatment with SPC (5 *μ*M) for 48 h.

Next, to clarify the role of YDJC in SPC-evoked EMT, we examined whether YDJC gene silencing and overexpression influence the SPC-evoked EMT in lung cancer cell lines. YDJC siRNA suppressed SPC-induced EMT (Figure 3(a), Fig.  S1). It also suppressed snail and slug expression in lung cancer cell lines (Figure 3(a), Fig.  S1). Conversely, YDJC overexpression induced EMT, snail, and slug expression, even without SPC treatment (Figure 3(a), Fig.  S1). YDJC siRNA suppressed and YDJC overexpression promoted SPC-induced loss of E-cadherin and enhanced expression of N-cadherin in H838 and H1299 cells (Figure 3(b), Fig.  S2). YDJC's involvement in SPC-induced loss of E-cadherin expression and enhanced expression of N-cadherin in A549 cells was proven by confocal microscopy (Figure 3(c)). Also, SPC-induced migration and invasion were inhibited by siRNA of YDJC and enhanced by overexpression of YDJC in lung cancer cell lines (Figure 3(d)). TGF-*β*1 could not induce changes of EMT markers such as E-cadherin and N-cadherin in YDJC stably knockdown A549 lung cancer cells (A549_shYDJC_) (Figure 3(e), Fig.  S3). These results suggest that YDJC might play a crucial role in SPC or TGF-*β*1-induced EMT.

YDJC is a member of the YDJC superfamily and has carbohydrate deacetylase activity, but no substrate has been identified in mammals so far [2, 19]. It is very mysterious how YDJC, a carbohydrate deacetylase, plays a role in EMT. First, we investigated whether YDJC is involved in EMT through enzymatic activity or interaction with other proteins regardless of enzyme activity. Luckily, aspartic acid at position 13 (D13) is considered a critical amino acid as a vital proton acceptor of deacetylase activity in YDJC [6, 19]. So, aspartic acid at position 13 (D13) of YDJC was changed into alanine (D13A) as the dominant negative form (YDJC_D13A_). The expressions of EMT markers such as E-cadherin and N-cadherin were changed by YDJC (wild type) overexpression even without SPC treatment. In contrast, YDJC_D13A_ overexpression failed to evoke changes in the expression of EMT markers (Figure 3(f), Fig.  S4). These results were confirmed by confocal microscopy (Figure 3(g)). Overexpression of YDJC_D13A_ was unable to promote SPC-promoted migration and invasion (Figure 3(h)). These results propose that YDJC's deacetylase activity is a critical factor in SPC-induced EMT.

### 3.4. CDC16 Suppresses YDJC-Induced EMT

**Figure 4:**
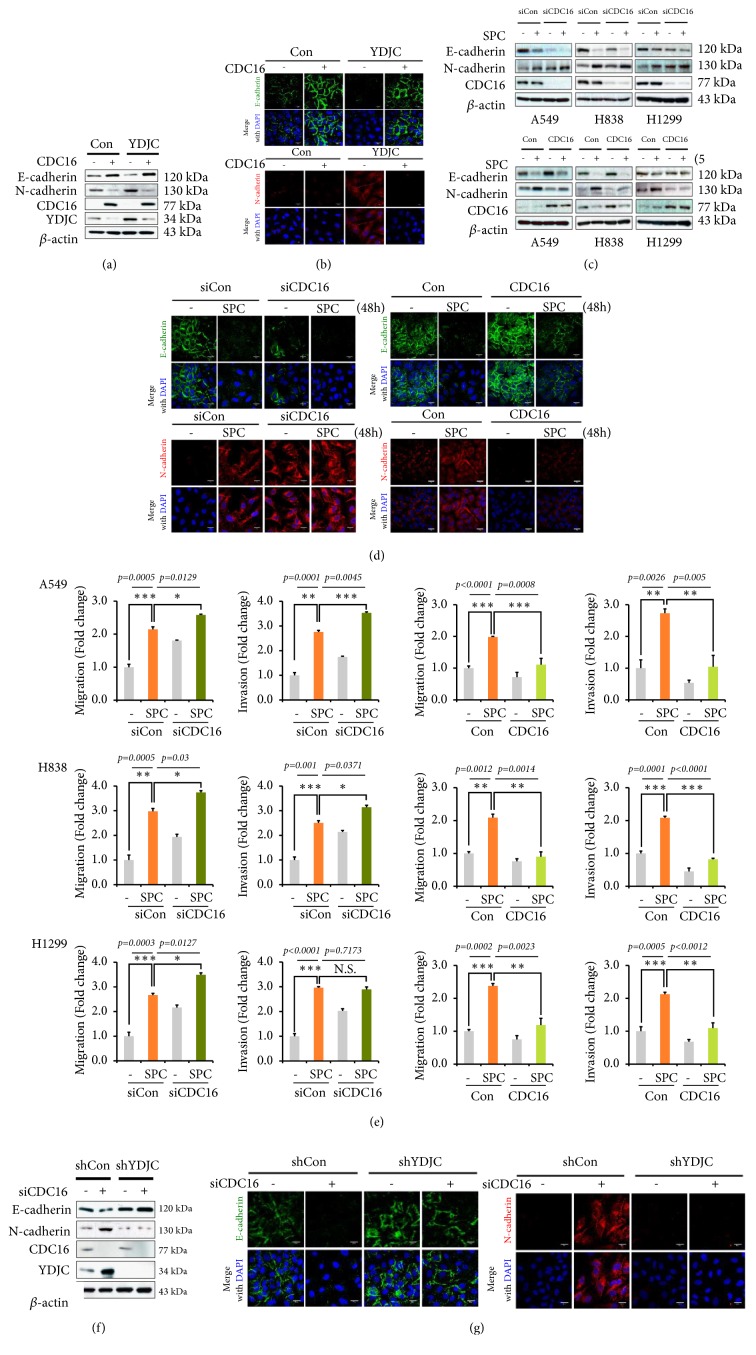
CDC16 regulates EMT. (a) Effect of CDC16 overexpression on SPC-induced EMT. CDC16 overexpression was done by transfecting A549 cells with the plasmid containing CDC16 and the empty control vector (4 *μ*g) followed by subsequent treatment with SPC (5 *μ*M) for 48 h. (b) Confocal microscopic analysis of the effect of CDC16 overexpression on SPC-induced EMT. (c) Effect of CDC16 siRNA (siCDC16) on SPC-induced EMT. Gene silencing of CDC16 was accomplished by transfecting A549, H838, and H23 cells with the CDC16 siRNA and control siRNA and stimulation with or without 5 *μ*M SPC for 48 h, respectively. (d) Confocal microscopic analysis of the effect of CDC16 siRNA (siCDC16) on SPC-induced EMT. (e) Effects of CDC16 siRNA and CDC16 overexpression on migration and invasion of A549 lung cancer cells. After siRNA of CDC16 and subsequent treatment with SPC, the A549, H838, and H1299 cells were added to the upper chamber of a Transwell insert to conduct the migration and invasion test. (f) Effects of CDC16 siRNA on changes of EMT markers in A549_shYDJC_ lung cancer cells. (g) Confocal microscopic analysis of the effects of CDC16 siRNA on changes of EMT markers in A549_shYDJC_ lung cancer cells. In (a), (c), and (f), cell lysates were analyzed by Western blotting.

Previously, we found that CDC16 directly binds YDJC and suppresses its action [6]. First, we investigated the effects of CDC16 expression in YDJC-induced EMT. CDC16 overexpression increased expression of E-cadherin and decreased N-cadherin expression in A549_YDJC_ cells (Figure 4(a), Fig.  S5). These results were proven by confocal microscopy (Figure 4(b)) and suggested that CDC16 blocked the action of YDJC on EMT in lung cancer cells. Then, we examined the effects of CDC16 siRNA on SPC-induced EMT. Interestingly, CDC16 gene silencing reduced E-cadherin expression and induced N-cadherin expression even without SPC treatment (Figure 4(c), Fig.  S6). However, overexpression of CDC16 reduced N-cadherin levels in the presence of SPC (Figure 4(c)). These results were also proven by confocal microscopy (Figure 4(d)). Furthermore, siCDC16 induced migration and invasion (Figure 4(e)). However, CDC16 overexpression mitigated SPC-induced migration and invasion (Figure 4(e)). Remarkably, in the YDJC stably knockdown A549 cell line, CDC16 siRNA failed to change the expression of EMT markers (Figure 4(f), Fig.  S7). These were confirmed by confocal microscopic analysis (Figure 4(g)). These data imply that CDC16 suppresses YDJC-induced EMT.

### 3.5. ERK2 Is Activated in siCDC16-Induced EMT

**Figure 5:**
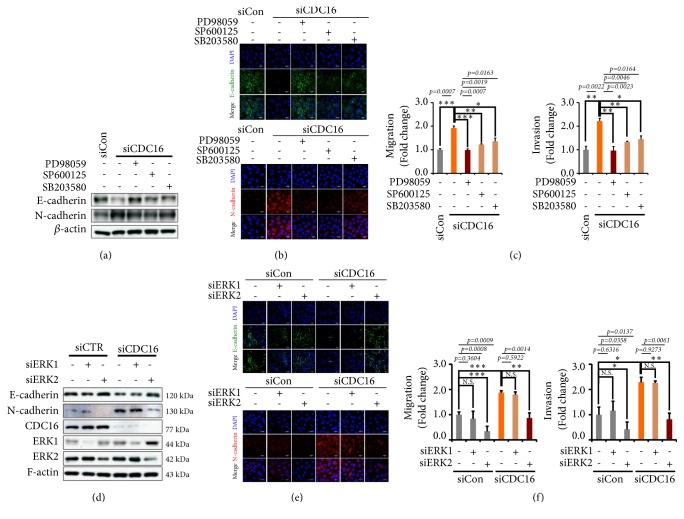
*ERK2 mediates siCDC16-induced EMT*. (a) MAP kinase inhibitors suppressed siCDC16-induced EMT in A549 cells. A549 cells were treated with or without MAP kinase inhibitors for 1 h in the presence of PD98059 (MEK inhibitor, 10 *μ*M), SP600125 (JNK inhibitor, 5 *μ*M), or SB203580 (p38 kinase inhibitor, 10 *μ*M). (b) Confocal microscopic analysis of the inhibitory effects of MAP kinase inhibitors on siCDC16-evoked EMT in A549 cells. (c) Inhibitory effects of MAPK inhibitors on siCDC16-evoked migration and invasion. After treatment with or without MAP kinase inhibitors for 1 h followed by siCDC16, the A549 cells were added to the upper chamber of the Transwell insert. (d) Effects of siERK1 and siERK2 on siCDC16-induced EMT. (e) Confocal microscopic analysis of the inhibitory effects of siERK1 and siERK2 on siCDC16-induced EMT in A549 cells. (f) The inhibitory effects of siERK1 and siERK2 on siCDC16-evoked migration and invasion.

ERK1/2 is reported to mediate YDJC-evoked K8 phosphorylation [6]. Therefore, we clarified whether ERK1/2 is also involved in siCDC16-induced EMT. MAP kinase inhibitors suppressed the siCDC16-induced loss of E-cadherin and reduced the expression of N-cadherin (Figure 5(a), Fig.  S8). The E-cadherin level was remarkably restored by PD98059, a MEK inhibitor, confirmed by confocal microscopy (Figure 5(b)). MAP kinase inhibitors also suppressed siCDC16-induced migration and invasion (Figure 5(c)). PD98059 significantly inhibited siCDC16-induced migration and invasion (Figure 5(c)). Then, we wanted to know whether ERK1 or ERK2 is involved in EMT. siERK2 suppressed the siCDC16-induced loss of E-cadherin expression and enhanced N-cadherin expression (Figure 5(d), Fig.  S9). These were confirmed by confocal microscopy (Figure 5(e)). siERK2 but not siERK1 suppressed siCDC16-induced migration and invasion (Figure 5(f)). These results imply that ERK2 mediates siCDC16-induced EMT of A549 lung cancer cells.

### 3.6. CDC16 Induces but YDJC Reduces Protein Phosphatase 2A (PP2A) Expression

**Figure 6:**
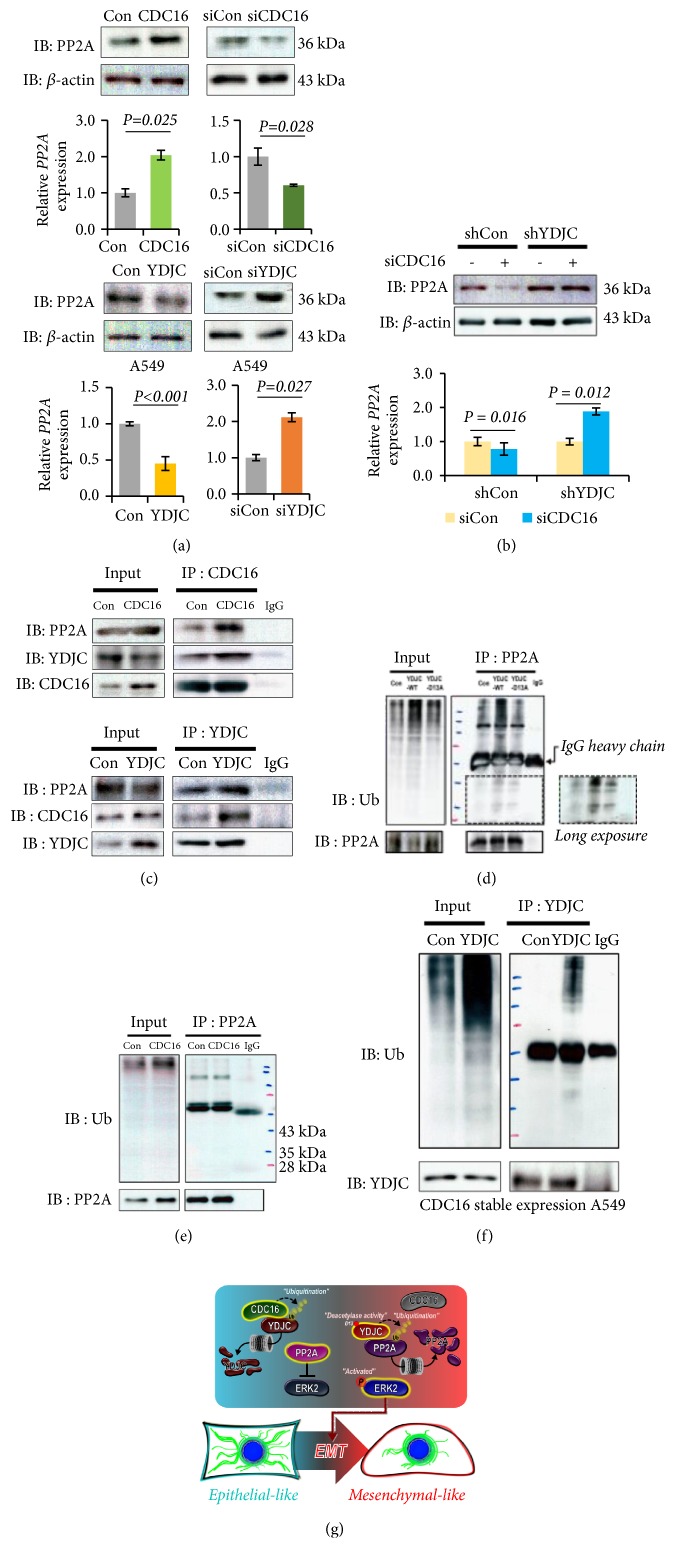
YDJC downregulates PP2A expression, and CDC16 reverses it. (a) CDC16 induces and YDJC suppresses PP2A expression. The expression levels of PP2A and* PP2A-C* mRNA levels in the Control (siCon) or YDJC-knockdown (siYDJC) or CDC16-knockdown (siCDC16) or YDJC-overexpressing (YDJC) or CDC16-overexpressing (CDC16)-A549 cells were analyzed using western blotting or qRT–PCR. (b) Effects of siCDC16 on PP2A expression in YDJC stably silenced A549 cell lines. The expression levels of PP2A and* PP2A-C* mRNA levels in the Control (siCon) or CDC16-knockdown (siCDC16)-YDJC stably silenced A549 cells were analyzed using western blotting or qRT–PCR. Mean± SD;* n*=3. All* P*-values were determined based on two-tailed Student's* t* test. In (a) and (b), A549 cells were transfected with the plasmid consisting of CDC16, YDJC, or an empty vector (4 *μ*g), respectively. Gene silencing of CDC16 or YDJC was carried out by transfecting the A549 cells with the indicated amounts of siCDC16, siYDJC, or control siRNA. Cell lysates were prepared, and the protein level was resolved to 8-12% SDS-PAGE. The expression level of PP2A was determined by western blotting; *β*-actin was used as an internal control. qRT-PCR analysis of* PP2A* expression in cDNA derived from the indicated cells.* GAPDH* is included as a loading control. (c) Binding of CDC16 to PP2A and YDJC. Immunoprecipitation with the CDC16 antibody (IP: CDC16) was carried out with the cell lysates of A549 cells overexpressed with CDC16 plasmids. The resulting immunocomplexes were analyzed with Western blotting with PP2A, YDJC, or CDC16 antibodies, respectively. (d) Stimulatory effects of YDJC on ubiquitination of PP2A. Immunoprecipitation with the PP2A antibody (IP: PP2A) was done using cell lysates of A549 cells overexpressed with wild-type YDJC_WT_ or YDJC_D13A_ plasmids. (e) Effects of CDC16 overexpression on PP2A ubiquitination. Immunoprecipitation with the PP2A antibody (IP: PP2A) was done using the cell lysates of A549 cells transfected with CDC16 plasmids. In d and e, the resulting immunocomplexes were detected by Western blotting with PP2A or ubiquitin (Ub) antibodies. (f) Effects of stably expressed CDC16 on YDJC ubiquitination in A549 cell lines. Immunoprecipitation with the YDJC antibody (IP: YDJC) was carried out using the cell lysates of A549 cells stably transfected with plasmids containing CDC16. The resulting immunocomplexes were detected by Western blotting with YDJC and Ub antibodies, respectively. (g) The proposed scheme of YDJC's mechanism of action.

In the previous report, we confirmed that YDJC binds to CDC16 which inhibits YDJC in SPC-induced K8 phosphorylation and reorganization via the ERK pathway [6]. However, we could not explain why ERK is activated in SPC or YDJC-evoked K8 phosphorylation and reorganization. Several research papers, including ours, suggest that PP2A negatively regulates the ERK pathway. Especially SPC-induced loss of EMP2 results in PP2A degradation through interaction with alpha4 leading to ERK activation [20, 21]. Therefore, we investigated the effects of YDJC and CDC16 on PP2A expression. By Western blot and qRT-PCR, we found that CDC16 induces but YDJC reduces PP2A expression (Figure 6(a), Fig.  S10). In contrast, gene silencing of CDC16 reduces PP2A levels, whereas gene silencing of YDJC induces PP2A levels (Figure 6(a)). Interestingly, in the A549 cell line with a stably knockdown of YDJC, the PP2A level was increased regardless of CDC16 siRNA (Figure 6(b), Fig.  S11).

We speculated that YDJC or CDC16 might affect ubiquitination of PP2A since CDC16 comprises a ubiquitin ligase in the APC/C complex [22]. So first, we examined whether CDC16 or YDJC directly binds to PP2A and found that YDJC and CDC16 bind to PP2A (Figure 6(c), Fig.  S12).

Remarkably, YDJC_WT_ overexpression increased PP2A ubiquitination compared with control or YDJC_D13A_ overexpression (Figure 6(d)), whereas CDC16 overexpression did not induce ubiquitination of PP2A (Figure 6(e)). These results suggest that PP2A ubiquitination is affected by YDJC but not CDC16 overexpression. Interestingly, YDJC was ubiquitinylated in CDC16 stably expressed A549 cells (Figure 6(f)). These results suggest that YDJC might induce ubiquitination of PP2A, but CDC16 might maintain PP2A expression by inducing ubiquitination of YDJC.

## 4. Discussion

In this study, we investigated whether SPC-induced YDJC expression promotes the progression of lung cancer via EMT. We found that YDJC induced EMT via ERK2 activation by PP2A downregulation through ubiquitination leading to the promotion of the progression of lung cancer and CDC16 reversed this by directly interacting with YDJC leading to ubiquitination. We previously reported that SPC or SPC-induced YDJC induces K8 phosphorylation and reorganization leading to migration and invasion of lung cancer cells [6]. These phenomena are involved in the change of viscoelasticity in cancer cells [17, 23, 24]. However, in the previous report, we could not confirm the role of YDJC in the progression of lung cancer in an in vivo mouse model. So we confirmed the involvement of YDJC in lung cancer progression using a lung cancer orthotopic mouse model (Figure 1). We also confirmed the activation of ERK2 and the possible involvement of PP2A by IHC staining (Figure 1(d)). Recent studies reported that continuous keratin phosphorylation and reorganization lead to loss of keratin [17], which is the hallmark of EMT [18]. So, we examined the possibility of EMT by IHC staining of EMT markers such as E-cadherin and N-cadherin (Figure 1(e)).

In this regard, we speculated that the EMT might be induced by long-term SPC treatment (48 h). In SPC-treated lung cancer cells, E-cadherin levels were downregulated, whereas N-cadherin and YDJC levels were upregulated (Figure 2), which was followed by increased migration and invasion (Figure 2(d)). These results strongly indicate that SPC promotes EMT in lung cancer cells.

From Figures 2 and 3, we showed that YDJC is induced and involved in SPC or TGF-*β*1-induced EMT. The involvement of YDJC in EMT induced by TGF-*β*, which is a well-known typical EMT inducer, suggests that involvement of YDJC in EMT might be a common mechanism of action of EMT induced by other EMT inducers including TGF-*β*1 and SPC.

How does YDJC drive EMT? Our data suggest that YDJC deacetylase activity is critical in SPC-induced EMT of human lung cancer cells although we do not know the exact mechanism of action of YDJC in EMT (Figures 3(f), 3(g), and 3(h)). Accordingly, it has been reported that the deacetylase activity of other proteins occurs in EMT. In the case of human renal epithelial cells, histone deacetylase activity modulated TGF-*β*1-induced EMT [25]. Besides, the H3K9 deacetylase activity of SIRT6 mediates EMT and metastasis in malignant human colon carcinoma [26]. Moreover, the blocking of histone deacetylase suppressed TGF-*β*1-induced EMT in hepatocytes [27]. These reports suggest that deacetylase activity plays an essential role in EMT. However, it is unknown whether this deacetylase activity in YDJC is limited to the removal of the carbohydrates' N-acetyl group or expands to the deacetylation of N-acetylated lysine in proteins.

We found that CDC16 might be a binding partner of YDJC [28]. Besides, we have previously shown that YDJC binds to CDC16, which suppressed YDJC's function [6]. Thus, we clarified the role of CDC16 on YDJC-induced EMT. Curiously, CDC16 gene silencing induced EMT, migration, and invasion, even without SPC treatment in lung cancer cell lines (Figure 4). Further, overexpression of CDC16 inhibited EMT, migration, and invasion (Figure 4). Moreover, in an YDJC stably knockdown A549 cell line, CDC16 gene silencing did not induce EMT in the absence of YDJC (Figure 4(f)). These results indicate that YDJC might mediate siCDC16-induced EMT and CDC16 may be an upstream suppressor of YDJC-induced EMT. Still, we do not know the mechanism of CDC16 involved in the suppression of YDJC expression. CDC16 might promote ubiquitination of YDJC since CDC16 comprises a ubiquitin ligase in the APC/C complex [22]. However, it is unknown whether the APC/C complex ubiquitinylates YDJC, although lysine 92 in human YDJC is ubiquitinylated [29].

As shown in a previous report, the ERK pathway mediates YDJC-induced keratin phosphorylation and reorganization [6]. Moreover, activated ERKs are involved in EMT [30, 31]. We found that CDC16, located upstream of YDJC, inhibits YDJC-induced EMT. We investigated whether ERK activation mediates CDC16 gene silencing-induced EMT and found that mitogen-activated protein kinase (MAPK) is involved in siCDC16-induced EMT. PD98059, which inhibits ERK phosphorylation acting as a MEK inhibitor, and SP600125, as a JNK inhibitor, prohibited siCDC16-evoked EMT, migration, and invasion of A549 cells (Figure 5). In particular, ERK signalling mediates siCDC16-induced EMT (Figure 5). Thus, subsequent studies need to focus on how CDC16 and YDJC regulate the activation of ERK. We found that ERK2 but not ERK1 mediates siCDC16-induced EMT (Figures 5(d), 5(e), and 5(f)). Several other studies about ERK2 involvement in EMT also support our findings [12, 32–34].

PP2A induces inactivation of phospho-ERK [16, 35, 36]. Overexpression of CDC16 induced PP2A expression, whereas overexpression of YDJC reduced PP2A expression (Figure 6(a)). In contrast, gene silencing of CDC16 reduced PP2A expression, whereas gene silencing of YDJC induced PP2A expression (Figure 6(a)). Remarkably, in the A549_shYDJC_ cell line, the PP2A level was increased by the presence or absence of CDC16 siRNA (Figure 6(b)). Furthermore, YDJC overexpression induced ubiquitination of PP2A. Besides, a YDJC deacetylase dominant-negative mutant (YDJC_D13A_) failed to induce PP2A ubiquitination (Figure 6(d)). Interestingly, the overexpression of CDC16 did not increase ubiquitination of PP2A (Figure 6(e)). These results indicate the possibility that YDJC enhances EMT via ubiquitination of PP2A, but CDC16 indirectly induces PP2A via enhancing ubiquitination of YDJC (Figure 6(g)).

How does YDJC increase the ubiquitination of PP2A? We do not know the relevant mechanism of action in YDJC's involvement in the ubiquitination of PP2A. However, we speculated the possible mechanism. Usually, acetylation of lysine prevents ubiquitination in the protein's lysine residue [37]. Therefore, if YDJC acts as a deacetylase of protein removal of the acetyl group of a lysine residue (lysine 41) in PP2A, YDJC might induce ubiquitination of lysine 41 in PP2A (Figure 6(g)). Based on these speculations, YDJC might increase the chance of the deacetylated lysine 41 in PP2A to be exposed to ubiquitinylation (Figure 6(d)). PP2A was ubiquitinylated by several ubiquitin ligases such as CRL4-DCAF1 and EDD E3 ubiquitin E3 ligases [38, 39]. Especially, progestin-induced EDD E3 ubiquitin ligase ubiquitinylates the PP2A catalytic subunit by interaction with alpha 4 which was already described in the degradation of PP2A during the SPC-induced loss of EMP2 [20, 21, 39].

In summary, we showed that YDJC promotes the progression of lung cancer in the orthotopic mouse model of lung cancer via EMT (Figure 1). We also proposed the novel mechanism of CDC16/YDJC/PP2A/ERK2 in lung cancer progression although it is necessary to confirm the proposed mechanism of the scheme (Figure 6(g)). Our data also suggest that YDJC might become an unusual target to prohibit lung cancer progression, although currently there are no reports of any compounds that inhibit YDJC.

## Data Availability

The data supporting the findings of our study are shown within this article.

## Supplementary Materials

Supplementary Materials Fig. 1S: quantification of E-cadherin, N-cadherin, and YDJC related to Figure 3(a). Fig. 2S: quantification of E-cadherin, N-cadherin, and YDJC related to Figure 3(b). Fig. 3S: quantification of E-cadherin, N-cadherin, and YDJC related to Figure 3(e). Fig. 4S: quantification of E-cadherin and N-cadherin related to Figure 3(f). Fig. 5S: quantification of E-cadherin, N-cadherin, and YDJC related to Figure 4(a). Fig. 6S: quantification of E-cadherin, N-cadherin, and YDJC related to Figure 4(c). Fig. 7S: quantification of E-cadherin and N-cadherin related to Figure 4(f). Fig. 8S: quantification of E-cadherin, N-cadherin, and YDJC related to Figure 5(a). Fig. 9S: quantification of E-cadherin, N-cadherin, and ERK1/2 related to Figure 5(d). Fig. 10S: quantification of PP2A related to Figure 6(a). Fig. 11S: quantification of PP2A related to Figure 6(b). Fig. 12S: quantification of PP2A related to Figure 6(c).
