# Supplementary material for: YDJC Induces Epithelial-Mesenchymal Transition via Escaping from Interaction with CDC16 through Ubiquitination of PP2A
Source: J Oncol. 2019 Aug 7;2019:3542537. doi: 10.1155/2019/3542537 (PMC6702825; doi:10.1155/2019/3542537)

# Supplementary Figure Legends

Fig S1. Quantification of E-cadherin, N-cadherin, and YDJC related to Figure 3a.

Fig. S2. Quantification of E-cadherin, N-cadherin, and YDJC related to Figure 3b.

Fig. S3. Quantification of E-cadherin, N-cadherin, and YDJC related to Figure 3e.

Fig. S4. Quantification of E-cadherin, and N-cadherin related to Figure 3f.

Fig. S5. Quantification of E-cadherin, N-cadherin, and YDJC related to Figure 4a.

Fig. S6. Quantification of E-cadherin, N-cadherin, and YDJC related to Figure 4c.

Fig. S7. Quantification of E-cadherin, and N-cadherin related to Figure 4f.

Fig. S8. Quantification of E-cadherin, N-cadherin, and YDJC related to Figure 5a.

Fig. S9. Quantification of E-cadherin, N-cadherin, and ERK1/2 related to Figure 5d.

Fig. S10. Quantification of PP2A related to Figure 6a.

Fig. S11. Quantification of PP2A related to Figure 6b.

Fig. S12. Quantification of PP2A related to Figure 6c.

# Supplementary Fig. 1S

Fig. 3a

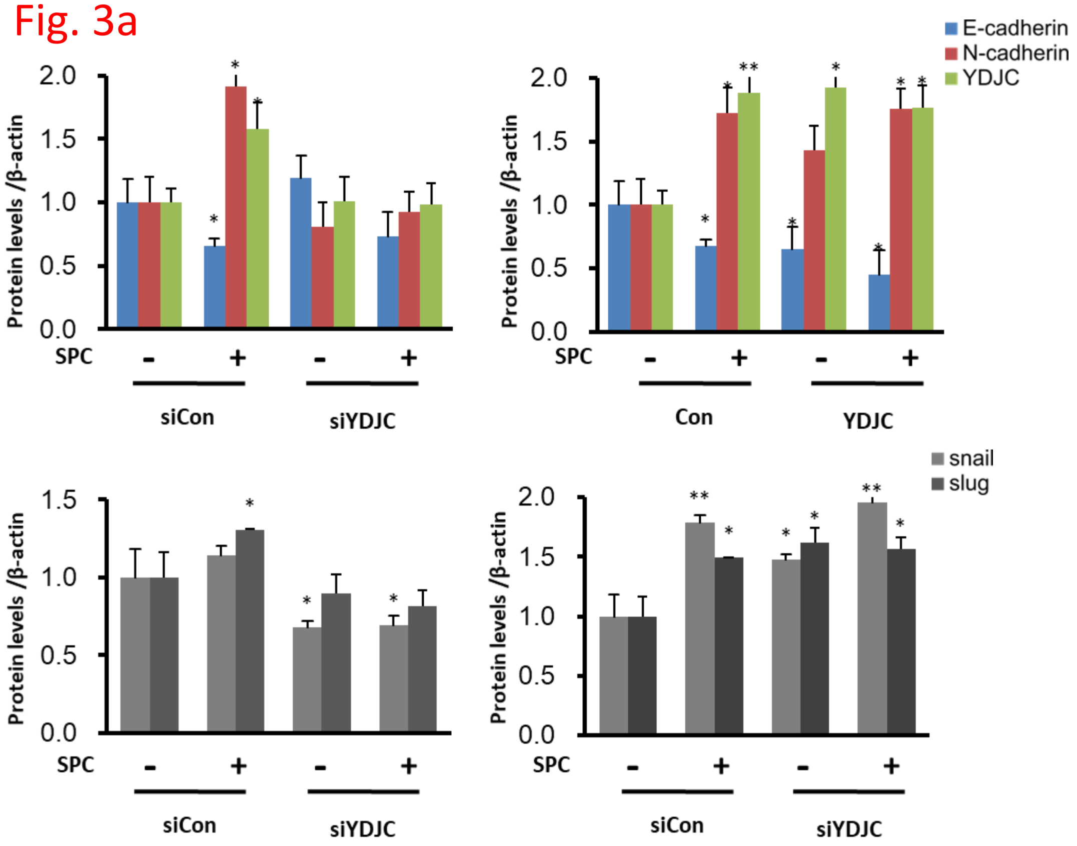


# Supplementary Fig. 2S

Fig. 3b

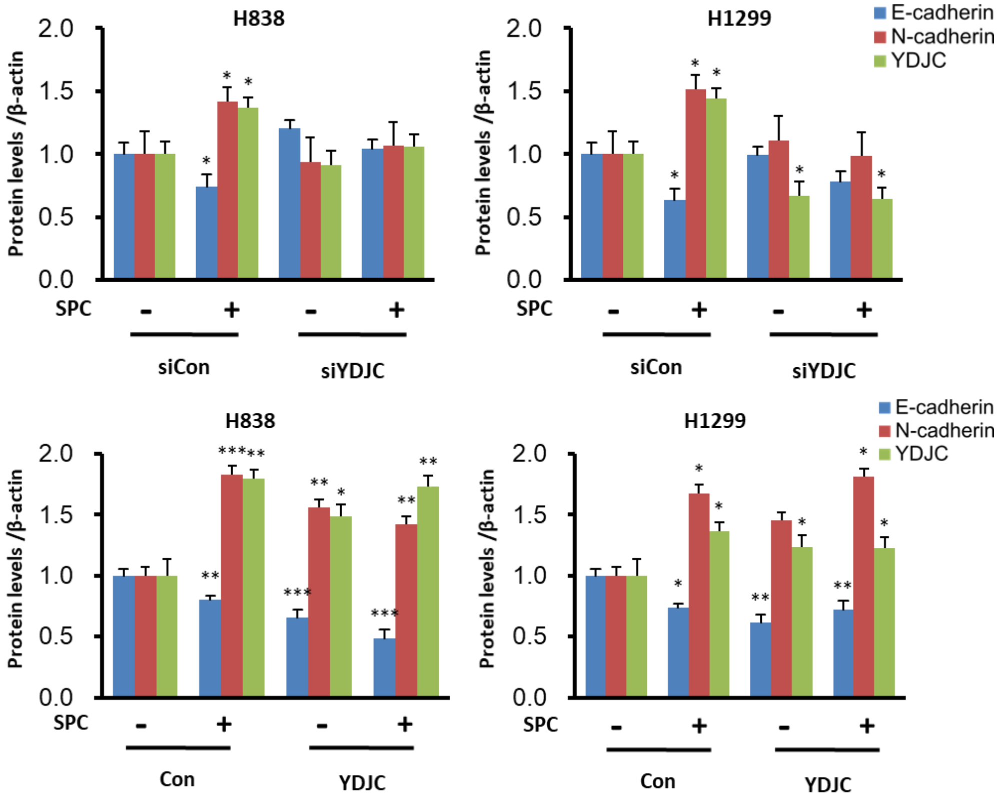

## Supplementary Fig. 3S

Fig. 3e

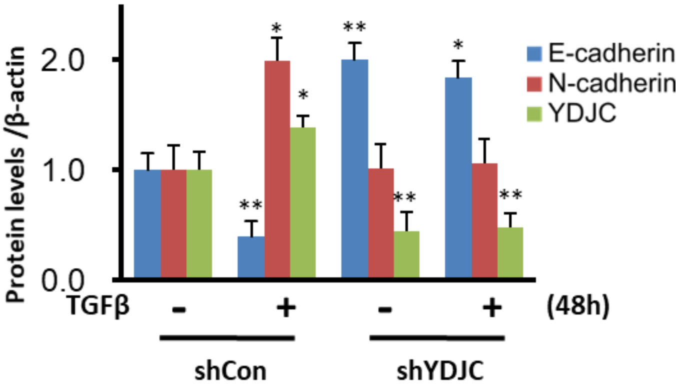


## Supplementary Fig. 4S

Fig. 3f

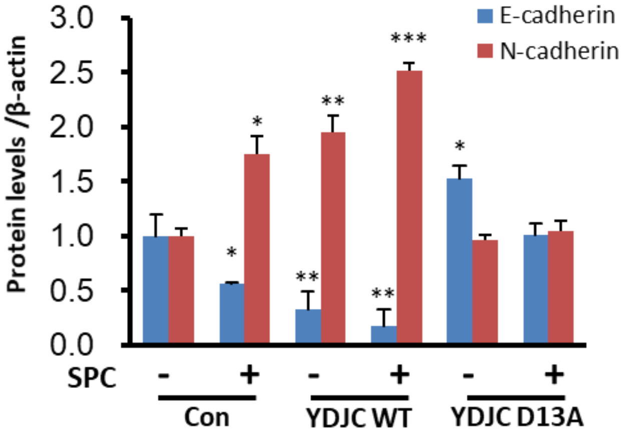


## Supplementary Fig. 5S

Fig. 4a

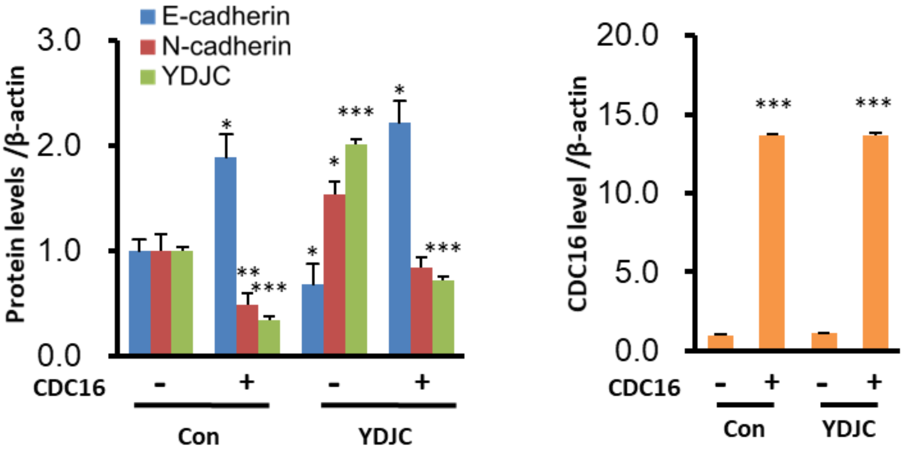

Supplementary Fig. 6S

Fig. 4c

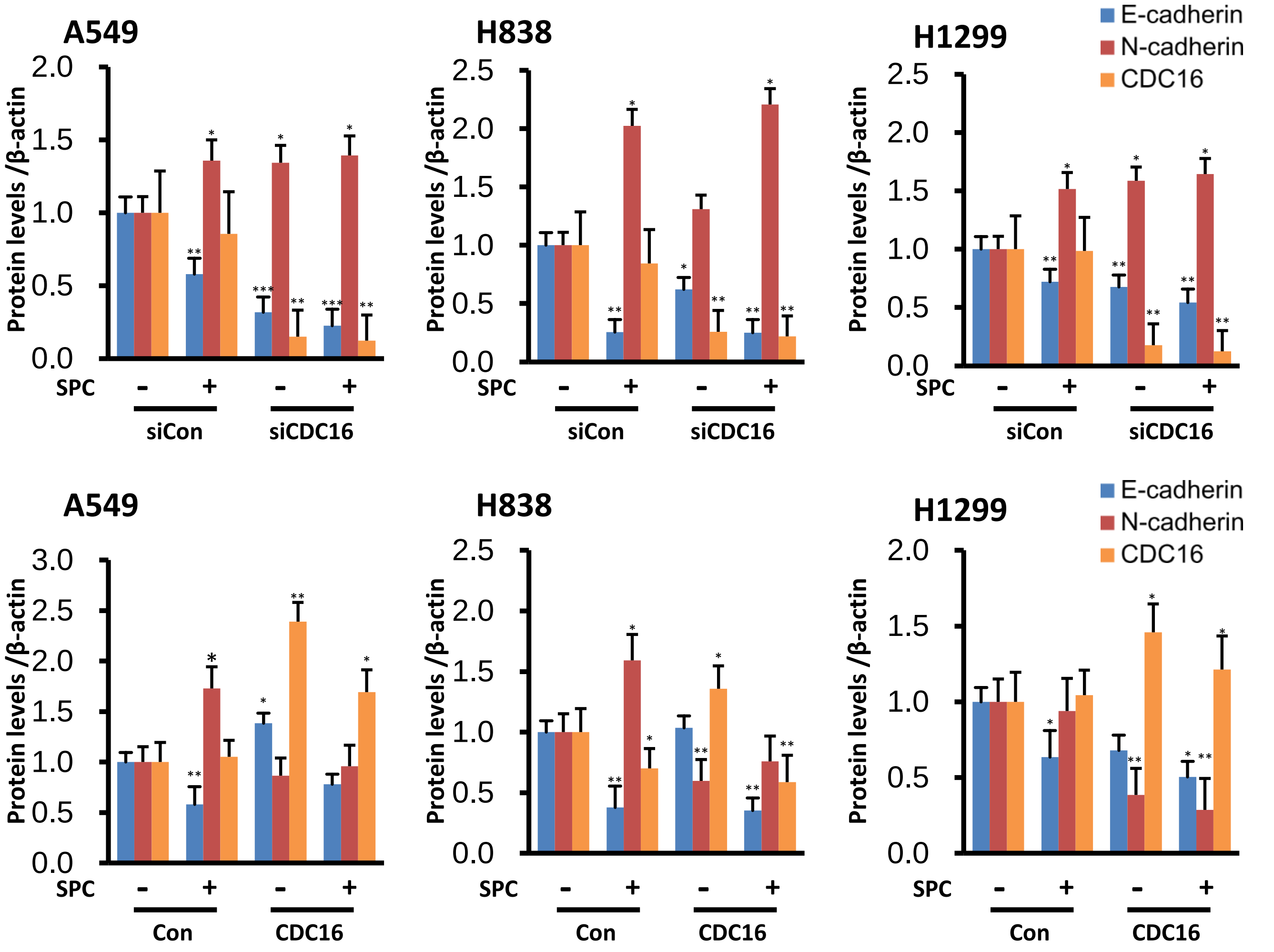


Supplementary Fig. 7S

Fig. 4f

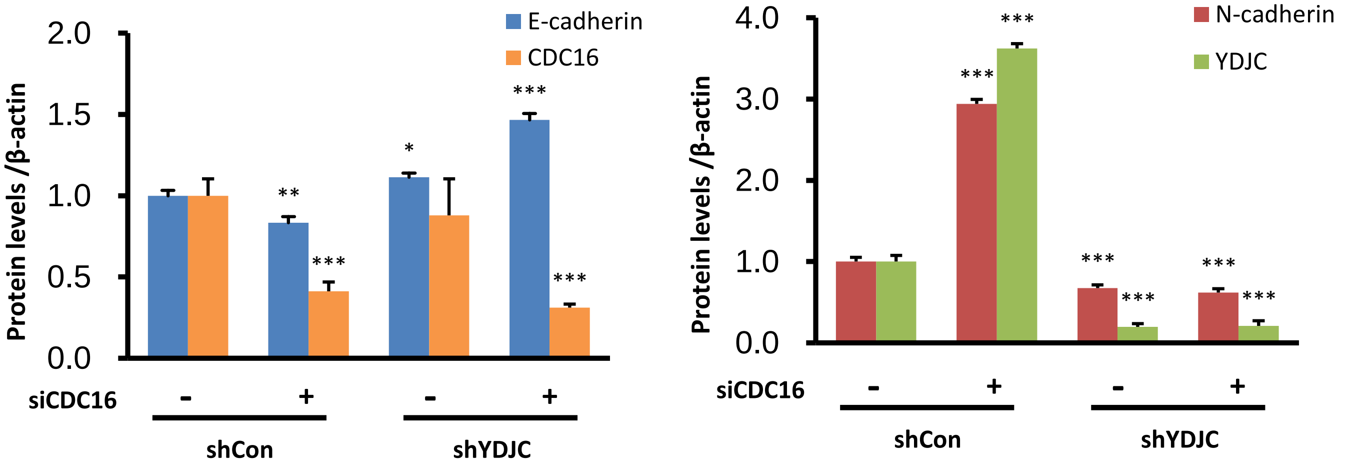

Supplementary Fig. 8S

Fig. 5a

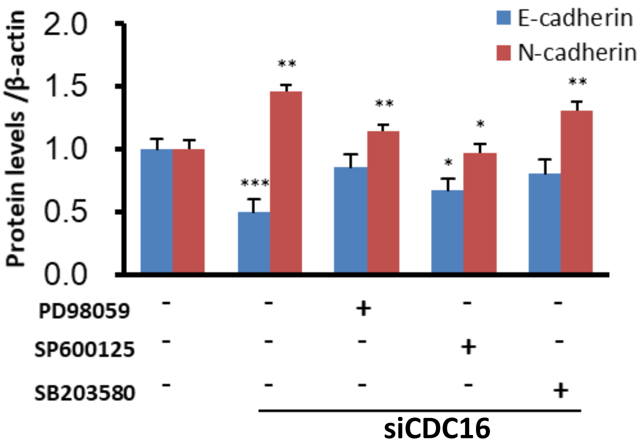


Supplementary Fig. 9S

Fig. 5d

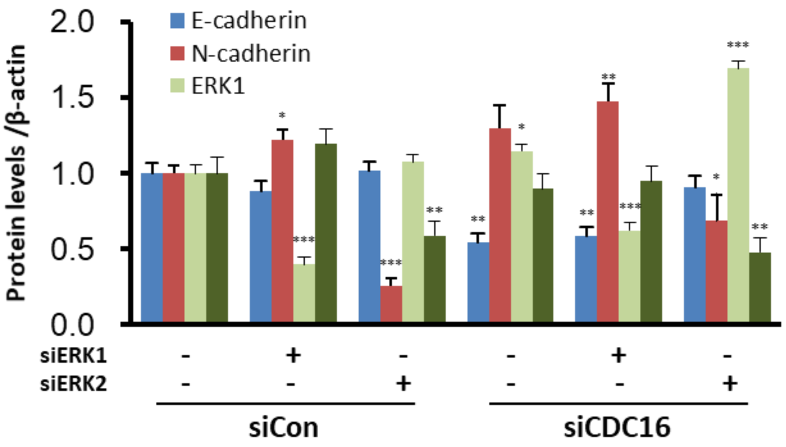


Supplementary Fig. 10S

Fig. 6a

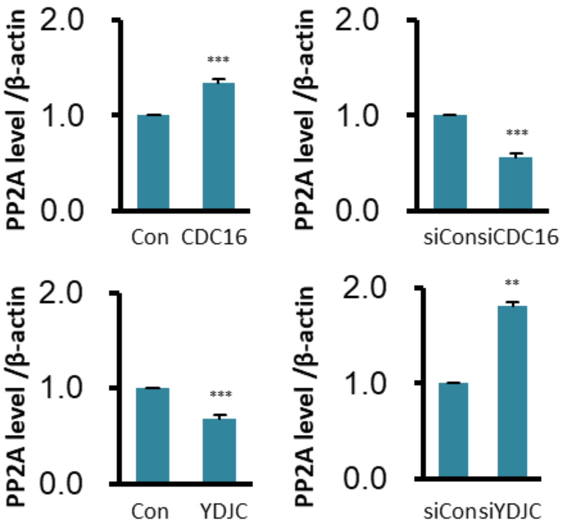


Supplementary Fig. 11S

Fig. 6b

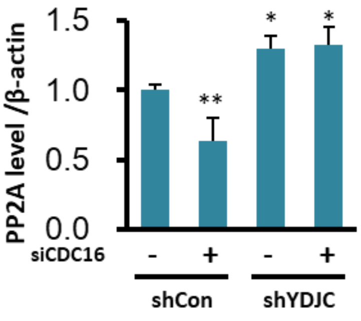

Supplementary Fig. 12S

Fig. 6c

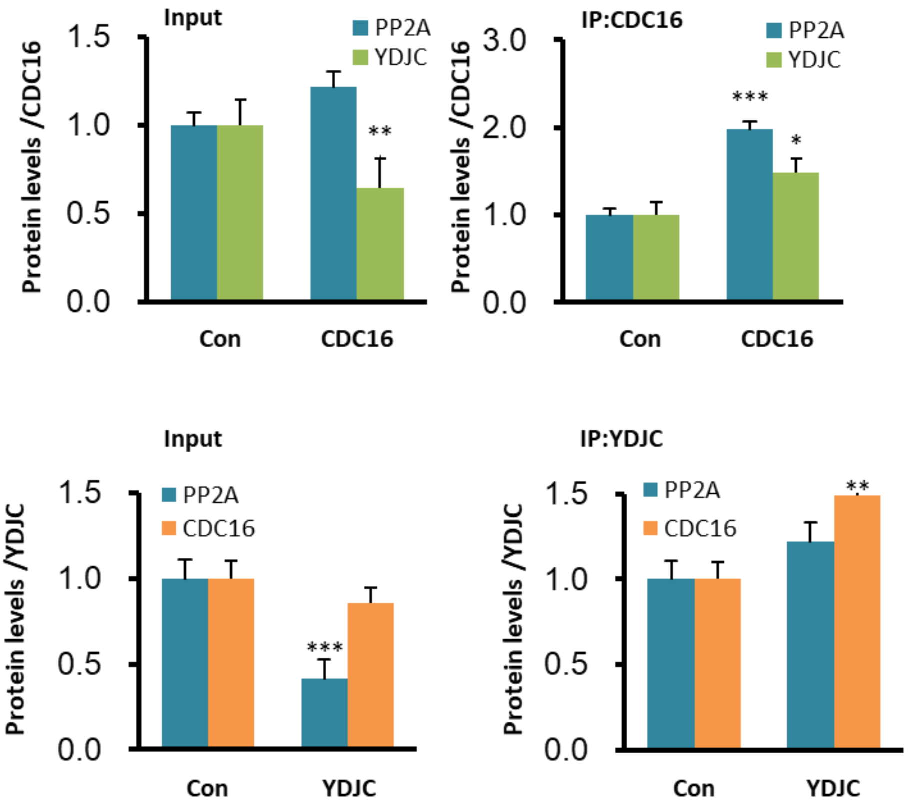

Supplement: Supplementary Materials — Fig. 1S: quantification of E-cadherin, N-cadherin, and YDJC related to Figure 3(a). Fig. 2S: quantification of E-cadherin, N-cadherin, and YDJC related to Figure 3(b). Fig. 3S: quantification of E-cadherin, N-cadherin, and YDJC related to Figure 3(e). Fig. 4S: quantification of E-cadherin and N-cadherin related to Figure 3(f). Fig. 5S: quantification of E-cadherin, N-cadherin, and YDJC related to Figure 4(a). Fig. 6S: quantification of E-cadherin, N-cadherin, and YDJC related to Figure 4(c). Fig. 7S: quantification of E-cadherin and N-cadherin related to Figure 4(f). Fig. 8S: quantification of E-cadherin, N-cadherin, and YDJC related to Figure 5(a). Fig. 9S: quantification of E-cadherin, N-cadherin, and ERK1/2 related to Figure 5(d). Fig. 10S: quantification of PP2A related to Figure 6(a). Fig. 11S: quantification of PP2A related to Figure 6(b). Fig. 12S: quantification of PP2A related to Figure 6(c). [file 3542537.f1.zip › 3542537.f1.pdf]
